# Pediatric phase I trial of oral sorafenib and topotecan in refractory or recurrent pediatric solid malignancies

**DOI:** 10.1002/cam4.598

**Published:** 2015-12-29

**Authors:** Damon R. Reed, Leo Mascarenhas, Kathleen Manning, Gregory A. Hale, John Goldberg, Jonathan Gill, Eric Sandler, Michael S. Isakoff, Tiffany Smith, Jamie Caracciolo, Richard M. Lush, Tzu‐Hua Juan, Jae K. Lee, Anthony M. Neuger, Daniel M. Sullivan

**Affiliations:** ^1^ Sarcoma DepartmentH. Lee Moffitt Cancer Center and Research InstituteTampaFlorida; ^2^ Chemical Biology and Molecular Medicine ProgramH. Lee Moffitt Cancer Center and Research InstituteTampaFlorida; ^3^ Adolescent and Young Adult ProgramH. Lee Moffitt Cancer Center and Research InstituteTampaFlorida; ^4^ Division of Hematology, Oncology, Blood and Marrow TransplantationDepartment of Pediatrics, Children's Hospital of Los Angeles and Keck School of MedicineUniversity of Southern CaliforniaLos AngelesCalifornia; ^5^ All Children's HospitalJohns Hopkins MedicineSt. PetersburgFlorida; ^6^ University of MiamiMiamiFlorida; ^7^ Children's Hospital at MontefioreAlbert Einstein College of MedicineBronxNew York; ^8^ Nemours Children's Cancer CenterJacksonvilleFlorida; ^9^ Connecticut Children's Medical CenterHartfordConnecticut; ^10^ Department of Diagnostic ImagingH. Lee Moffitt Cancer Center and Research InstituteTampaFlorida; ^11^ Department of Biostatistics and BioinformaticsH. Lee Moffitt Cancer Center and Research InstituteTampaFlorida; ^12^ Translational Research CoreH. Lee Moffitt Cancer Center and Research InstituteTampaFlorida; ^13^ Department of Blood and Marrow TransplantationH. Lee Moffitt Cancer Center and Research InstituteTampaFlorida

**Keywords:** Combination, pediatric cancer, phase I, sarcoma, sorafenib, topotecan

## Abstract

Targeted kinase inhibitors and camptothecins have shown preclinical and clinical activity in several cancers. This trial evaluated the maximum tolerated dose (MTD) and dose‐limiting toxicities of sorafenib and topotecan administered orally in pediatric patients with relapsed solid tumors. Sorafenib was administered twice daily and topotecan once daily on days 1–5 and 8–12 of each 28‐day course. The study utilized a standard 3 + 3 dose escalation design. Three dose levels (DL) were evaluated: (1) sorafenib 150 mg/m^2^ and topotecan 1 mg/m^2^; (2) sorafenib 150 mg/m^2^ and topotecan 1.4 mg/m^2^; and (3) sorafenib 200 mg/m^2^ and topotecan 1.4 mg/m^2^. Pharmacokinetics were ascertained and treatment response assessed. Thirteen patients were enrolled. DL2 was the determined MTD. Grade 4 thrombocytopenia delaying therapy for >7 days was observed in one of six patients on DL2, and grade 4 neutropenia that delayed therapy in two of three patients on DL3. A patient with preexisting cardiac failure controlled with medication developed a transient drop in the left ventricular ejection fraction that improved when sorafenib was withheld. Sorafenib exposure with or without topotecan was comparable, and the concentration‐time profiles for topotecan alone and in combination with sorafenib were similar. One objective response was noted in a patient with fibromatosis. We determined MTD to be sorafenib 150 mg/m^2^ twice daily orally on days 1–28 combined with topotecan 1.4 mg/m^2^ once daily on days 1–5 and 8–12. While these doses are 1 DL below the MTD of the agents individually, pharmacokinetic studies suggested adequate drug exposure without drug interactions. The combination had limited activity in the population studied.

## Introduction

Although outcomes for pediatric cancer patients have steadily improved with the introduction of systemic chemotherapy and through carefully conducted clinical trials in the 1960s through 1990s, cure rates for advanced‐stage, solid tumors have unfortunately plateaued over the past decade. Many single‐agent phase I trials have demonstrated safety and defined a dose for further study in pediatrics, but incorporating these agents in front‐line protocols has often remained a challenge 1. Osteosarcoma, Ewing sarcoma, neuroblastoma, rhabdomyosarcoma, and Wilms tumor are treated with combinations of 2–10 chemotherapeutic agents in the front‐line setting 2, 3, 4, 5. We had previously explored emerging targeted therapies along with selected cytotoxic agents across 2 osteosarcoma, 2 Ewing sarcoma, and a single rhabdoid tumor cell line to help develop early‐phase clinical trials 6. Among the combinations with additive or synergistic effects were topotecan and sorafenib.

Sorafenib, a broad multikinase inhibitor affecting the serine/threonine kinases c‐Raf and B‐Raf and the receptor tyrosine kinases RET, Flt‐3, and c‐Kit at nanomolar concentrations 7, has demonstrated efficacy in pediatric preclinical cell models with a median IC50 of 4.3 *μ*mol/L 8 and has been studied in hundreds of clinical trials 9. Sorafenib is currently approved by the Food and Drug Administration for hepatocellular carcinoma, renal cell carcinoma, and dedifferentiated thyroid cancer. Pediatric investigations of sorafenib alone and in combination have been reported since the current trial's inception, including combination trials in leukemia and solid tumors, which demonstrated tolerability and promising activity 10, 11. In a phase I single‐agent pediatric trial, sorafenib demonstrated good tolerability with doses similar to adult dosing by body‐surface area and dose‐limiting toxicities (DLTs) of rash and hypertension 12. Sorafenib has been studied in plexiform neurofibromas without clear efficacy and induced a response in a ventilation‐dependent patient with papillary thyroid cancer, leading to maintained disease remission after further therapy 13, 14.

Topotecan has established efficacy as a single agent and in combination in a variety of pediatric malignancies, including germ cell tumors, Wilms tumor, neuroblastoma, acute lymphoblastic leukemia, central nervous system malignancies, and sarcomas 15, 16, 17, 18, 19, 20, 21, 22, 23. Topotecan is currently being investigated in a randomized phase III Ewing sarcoma trial (NCT01231906) and as part of standard induction therapy for higher‐risk neuroblastoma patients. Topotecan is also commonly used alone and in combination with other cytotoxic chemotherapies such as cyclophosphamide for a broad spectrum of malignancies 23, 24. Recently, topotecan was combined with targeted agents in ovarian cancer, although with toxicity limiting maximal therapy delivery and modest activity 25. The broad potential and anecdotal reported activities of both agents in a variety of pediatric malignancies, together with our preclinical data in sarcoma cell lines, provided the rationale to investigate this combination in a clinical trial.

Our primary objective was to establish the maximum tolerated dose (MTD) for this combination. We started based on previously tested dose levels (DLs) at 2 levels below the topotecan MTD and 1 DL below the sorafenib single agent MTD. Secondary objectives were to describe toxicities, characterize pharmacokinetics (PK) of topotecan and sorafenib, apply Response Evaluation Criteria in Solid Tumors (RECIST) 1.1 to measure response after even‐numbered cycles, and determine time to progression (TTP) for all patients and compare TTP on study and TTP on previous regimen. Additionally, all therapy was delivered orally, allowing for minimal time spent in clinics and hospitals.

## Methods

### The sunshine project consortium

The Sunshine Project is multi‐institutional consortium funded by The Pediatric Cancer Foundation, with Moffitt Cancer Center as the coordinating center responsible for statistics, initial scientific review and Institutional Review Board approval, and regulatory aspects of the trial. Patients were enrolled at member sites only with 6 sites enrolling patients.

### Patient eligibility

Patients aged 3–18 years with relapsed or refractory solid tumor malignancies; including central nervous system malignancies and fibromatosis and previously treated with chemotherapy were eligible. Patients needed to have radiologic evidence of disease and were preferred, but not required, to have measurable disease using RECIST 1.1, a life expectancy of at least 12 weeks, no known curative therapy, a Karnofsky or Lansky score ≥50, recovered from prior therapy, no previous sorafenib therapy, >6 months since prior topotecan, >3 weeks since last myelosuppressive therapy, >7 days since filgrastim and >14 days since pegfilgrastim, >21 days or 4 half‐lives (whichever is greater) since biologic agent, ≥4 weeks since completion of local palliative irradiation, ≥3 months from prior Total Body Irradiation, craniospinal irradiation, or ≥50 Gy radiation of pelvis, ≥6 weeks for substantial bone marrow radiation, ≥3 months since Stem Cell Transplant and no evidence of active graft versus host disease along with transfusion and growth factor independence, adequate bone marrow function (absolute neutrophil count ≥1500/*μ*L, platelet count ≥100,000/*μ*L, and hemoglobin ≥10 gm/dL), normal serum creatinine for age or a glomerular filtration rate ≥60 mL/min/1.73 m^2^, bilirubin ≤ upper limit of normal (ULN), alanine transaminase ≤ULN, and all clinically significant chemistries grade 1 or less with the exclusion of alkaline phosphatase, uric acid, aspartate transaminase, and lactate dehydrogenase, prothrombin time and partial thromboplastin time ≤1.5 X ULN, 12‐lead electrocardiogram with corrected QTc <450 msec, either shortening fraction ≥28% or left ventricular ejection fraction ≥50%, systolic and diastolic blood pressure ≤95% for age and gender (antihypertensive management allowed), no ongoing cardiac dysrhythmias ≥grade 2, atrial fibrillation of any grade, unstable angina, symptomatic congestive heart failure, or myocardial infarction, resting pulse oximetry of ≥92% and no dyspnea at rest. Patients with known bone marrow metastatic disease were eligible but could not be refractory to red blood cell or platelet transfusion. Women who were pregnant or lactating, patients with nonhealing wounds/ulcers or bone fractures, and patients with a history of organ allograft were excluded. The Internal Review Board for each participating institution approved the protocol, and written informed consent and assent were obtained according to local institutional guidelines.

### Drug administration

The starting dose of sorafenib was 150 mg/m^2^ twice daily on DLs 1 and 2 on days 1–28, except in cycle 1 when it was given on days 2–28. Dose escalation for sorafenib occurred at DL 3 to the maximum planned dose of 200 mg/m^2^ (Table 1). Intra‐patient dose escalation was not allowed on this study. Sorafenib was supplied by Bayer Pharmaceuticals (Bayer HealthCare Pharmaceuticals, Wayne, NJ) as 50 mg tablets. Total daily dose for patients was rounded to the nearest 50 mg tablet size based on patient BSA (a dosing nomogram was used to minimize inter‐patient dosing variability). Patients ingested tablets with clear liquids (2–4 ounces for children <12 and 4 ounces for ≥12 years) and while dispersion in liquid was allowed, all patients swallowed tablets.

**Table 1:** Dose escalation schema and dose‐limiting toxicities

Dose level	Number of patients entered	Number of evaluable patients	Number of patients with dose‐limiting toxicity	Type of toxicity (*n*)
Topotecan 1.0 mg/m^2^ per day + Sorafenib 150 mg/m^2^ per twice daily	3	3	0	NA
Topotecan 1.4 mg/m^2^ per day + Sorafenib 150 mg/m^2^ per twice daily	6	6	1	Platelet count decreased (1)
Topotecan 1.4 mg/m^2^ per day + Sorafenib 200 mg/m^2^ per twice daily	3	3	2	Neutrophil count decreased (2)

The starting dose of topotecan at DL 1 was 1.0 mg/m^2^ on days 1–5 and 8–12 of each cycle. Dose escalation for topotecan started with DL 2–1.4 mg/m^2^ and continued on DL 3 (Table 1). Topotecan was commercially available from multiple suppliers in either a capsule (0.25 mg or 1 mg) form or reconstituted liquid formulation, with dose rounding to the nearest 0.25 mg. The capsules could not be chewed, crushed, or divided and were swallowed whole. The reconstituted liquid formation of topotecan was supplied as a lyophilized, light‐yellow powder in vials containing 4 mg of topotecan (as the base), which were reconstituted with 4 mL bacteriostatic water yielding 1 mg/mL solution of topotecan. Vials were dispensed in light protective bags and kept refrigerated. The reconstituted formulation was drawn up in oral syringes to the prescribed volume and administered to the patient immediately after reconstitution. The process was observed by the clinic staff on day 1 of the first cycle and then allowed unsupervised once the family demonstrated a clear understanding of the dosing. Eight ounces of water were used to rinse and swallow after the topotecan was administered.

### Study design

This dose‐escalation study used a standard 3 + 3 design, with up to six patients being enrolled on a DL. Enrollment to subsequent DLs was determined by the number of enrolled patients, the number with DLTs, and the number at risk for DLTs.

Toxicity was graded according to the Common Terminology Criteria for Adverse Events version 4.0 (http://ctep.cancer.gov). Hematologic DLTs were defined during cycle 1 only as grade 4 thrombocytopenia (platelet count <25,000/*μ*L) or grade 4 neutropenia (<500/*μ*L) lasting >7 days that was attributable to sorafenib or topotecan. Non‐hematologic DLTs were defined as any grade 4 nonhematological toxicity or grade 3 nonhematological toxicity with the following exceptions: grade 3 nausea/vomiting of <5 consecutive days with appropriate anti‐emetic therapy, grade 3 transaminase levels that returned to baseline levels within 7 days of study drug cessation, grade 3 fever/infection lasting <7 days, grade 3 hypocalcemia, hypokalemia, hypophosphatemia, and/or hypomagnesemia unresponsive to oral supplementation (defined as return to ≤grade 1), and asymptomatic chemical pancreatitis with elevations of amylase or lipase that returned to ≤grade 1 before meeting off‐study criteria. Non‐hematologic DLTs were also defined as any adverse event considered intolerable by the patient/family and requiring treatment interruption >14 days and any adverse event requiring interruption of study drug for >7 days and that recurred upon drug reintroduction. Dose‐limiting hypertension was defined as systolic or diastolic blood pressure >25 mmHg above the 95th percentile for age, height, and gender confirmed by repeated measurement for >14 consecutive days, as well as any grade 4 hypertension. The MTD was the DL immediately below the DL at which 2 or more patients in a DL experienced a DLT.

Treatment response was assessed per RECIST 1.1. Central review occurred at the end of study, but decisions regarding patient therapy were made in real time by treating physicians.

Patients were evaluated within 28 days before the start of study, at the completion of cycle 2, and at the end of each even‐numbered cycle if presenting with stable disease or better.

### Pharmacokinetic studies

#### Sample collection

Blood samples for sorafenib PK studies were collected on cycle 1, day 28 and cycle 2, day 1 at hours 0 (pre‐dose) and 1, 3, 5, and 8 h following the first dose of sorafenib, centrifuged at 1250*g* for 5 min, and frozen at −20°C until analysis. Blood samples for topotecan PK were collected on cycle 1, day 1 and cycle 2, day 1 at hours 0 (pre‐dose) and 1, 3, 5, and 8 h post‐dose and centrifuged at 1250*g* for 4 min. Exactly 400 *μ*L of the upper plasma layer was transferred into prelabeled cryovials, with one set containing reagents specific for assaying for total topotecan and another for assaying for lactone topotecan, and frozen at −20°C until analysis.

#### Analytical methodology

A liquid chromatography‐tandem mass spectrometry method validated under ICH/Food and Drug Administration guidelines was used to determine levels of sorafenib and was adapted from a previously published method 26. Plasma samples were analyzed by protein precipitation. Calibration curves, linear from 5 to 2500 ng/mL with an *R* ^2^ of >0.99, were generated for each run, with patient sample concentrations back‐calculated from the corresponding regression line.

Topotecan was measured by high‐performance liquid chromatography with fluorescence detection 27, validated under the same guidance, after protein precipitation, as previously described. The calibration curve was linear from 0.125 to 50 ng/mL, with the regression meeting acceptable criteria, with patient samples calculated as described above.

#### Pharmacokinetic analysis

Plasma concentration‐time data for both drugs were analyzed by noncompartmental methods using Phoenix WinNonlin 6.3 (Pharsight Corp., Mountain View, CA). The following steady‐state characteristics for sorafenib were determined: AUC_0‐8 h_, *C*_max_, *T*_max_, *C*_min_, *T*_min_, and *C*_avg_. For topotecan, when available, half‐life was determined along with AUC_0‐inf_, *C*_max_, *T*_max_, clearance, and volume of distribution.

## Results

Thirteen patients were enrolled between October 2013 and December 2014 across six sites. One patient withdrew consent before starting therapy, with the remaining 12 patients evaluable for toxicity. Patients had a median of two prior lines of therapy with a range of 1–4 prior lines of therapy (Table 2).

**Table 2:** Characteristics of evaluable patients (*n* = 12)

	Number (%)
Age, median (range)	13 years (8–18 years)
Sex	
Male	8 (66.7)
Female	4 (33.3)
Diagnosis	
Embryonal rhabdomyosarcoma	1 (8.3)
Ewing sarcoma	3 (25)
Fibromatosis	2 (16.7)
Neuroblastoma	1 (8.3)
Neuroendocrine carcinoma	1 (8.3)
Osteosarcoma	4 (33.3)
Prior therapy	
Chemotherapy regimens, median (range)	2 (1–4)
Radiotherapy (number of patients)	7
Bone marrow transplant (number of patients)	2
Race	
White	7 (58.3)
Asian	0 (0)
American Indian or Alaska Native	0 (0)
Black or African American	2 (16.7)
Unknown	3 (25)
Ethnicity	
Non‐Hispanic	7 (58.3)
Hispanic	5 (41.7)

### Toxicity

Three DLs were evaluated without a need for de‐escalation (Table 1). There were no deaths related to toxicity. DLTs were hematologic, including thrombocytopenia and neutropenia of defined duration over 7 days (Table 1). The MTD was reached at DL 2 with 2 of 3 patients experiencing DLTs at DL 3. Table 3 shows additional toxicities of at least grade 3 and possibly attributed to either sorafenib or topotecan and the maximal grade across all cycles for an individual patient is listed once. An osteosarcoma patient had a change in cardiac function that occurred during cycle 2, which was thus not considered a DLT. This patient had prior doxorubicin therapy to a cumulative dose of 450 mg/m^2^ and was on digoxin and lisinopril with the study entry ejection fraction meeting criteria for inclusion at 50.6% by echocardiogram. Due to poor cardiac medication compliance, it dropped to 37% during cycle 1 and rebounded to over 50% when digoxin and lisinopril were restarted. Sorafenib was also withheld during this time. When sorafenib was restarted during cycle 2, the ejection fraction again fell to grade 3 and the investigator, after consulting with cardiology, felt that the risk of continuing therapy outweighed the potential benefit and patient was taken off study. A single patient experienced grade 2 radiation recall, attributed to either topotecan or sorafenib and responded to topical therapy and withholding sorafenib and/or topotecan for 7 days during all cycles. During some of the cycles, the radiation recall occurred in mid‐cycle, requiring holding just sorafenib; in the other cycles, the radiation recall occurred at the end of the cycle, requiring delay in initiating the next cycle by 7 days 28. Severe treatment‐related adverse events included 3 instances of febrile neutropenia admissions and 2 admissions for blood product transfusion when outpatient facilities were not available.

**Table 3:** Toxicities (grade 3 or greater) observed in evaluable patients and attributed to at least possibly related to sorafenib or topotecan

Toxicity type	Grade 3	Dose level (*n*)	Grade 4	Dose level (*n*)
Alanine aminotransferase increased	2	2, 3	0	
Anemia	5	1, 2(4)	0	
Ejection fraction decreased	1	1	0	
Febrile neutropenia	3	2, 3(2)	0	
Hypertension	1	3	0	
Hypokalemia	1	2	0	
Nausea	1	2	0	
Neutrophil count decreased	3	1, 2, 3	7	1, 2(4), 3(2)
Platelet count decreased	1	2	10	1(2), 2(5), 3(3)
Radiation recall reaction (dermatologic)	1	3	0	
Vomiting	1	2	0	
Weight loss	1	1	0	

### Responses

Two patients with DLTs came off study before completing cycle 2, and both had clinical progression during the follow‐up period. Two patients had bone‐only disease and thus were not evaluable by RECIST 1.1, and another patient came off study before the first disease evaluation during cycle 2 due to physician choice. Thus, seven patients were evaluable for disease response. Of the 7 patients who could be evaluated for RECIST 1.1 response, 1 patient with fibromatosis showed radiologic partial response; this patient continues on the 8th cycle of therapy as of the data cut‐off. The other 6 patients with evaluable disease received 1–4 cycles of therapy with 5 of them receiving 2 cycles and with progressive disease at this first evaluation. The 2 patients with bone‐only disease had Ewing sarcoma and received 1 and 4 cycles before progression. TTP ranged from 11 to 111 days on study in the nonresponders (Table S1). Refractory disease, defined as <3 months between completion of prior therapy and enrollment was present in 6 of 7 evaluable patients. In the 7 evaluable patients, only the patient with fibromatosis had a TTP that was longer than the prior regimen.

### Pharmacokinetics

**Figure 1:**
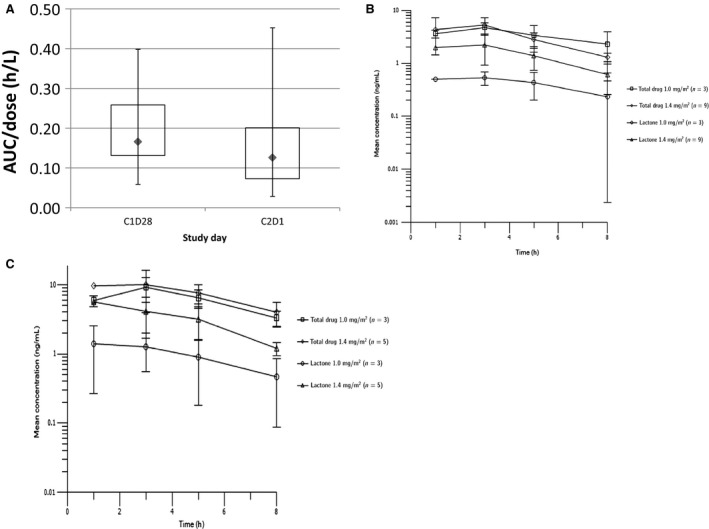
(A) Dose‐normalized sorafenib area under the curve across all dose levels (DLs) on cycle1, day 28 (administered alone) and cycle 2, day 1 (administered with topotecan). (B) Cycle 1, day 1 topotecan mean concentration (±SD) versus time by DL given without sorafenib. (C) cycle 2, day 1 topotecan mean concentration (±SD) versus time by DL, administered while sorafenib at steady state.

Nine patients were evaluable for sorafenib PK on cycle 1, day 28 and 7 patients on cycle 2, day 1. As shown in Table 4, steady‐state kinetics varied widely for both groups, independent of dose or in combination with topotecan. Overall, when AUC is dose normalized, there are comparable amounts of exposure to sorafenib with and without exposure to topotecan (Fig. 1A).

**Table 4:** Sorafenib and topotecan pharmacokinetic parameter estimates

	Sorafenib					
	*T*_max_ (h)	*C*_max_ (*μ*g/mL)	*T*_min_ (h)	*C*_min_ (*μ*g/mL)	*C*_avg_ (*μ*g/mL)	AUC_(0‐8 h)_ (h × *μ*g/mL)
Cycle 1, day 28, 150 mg/m^2^ (*n* = 8)	4.0 ± 3.3	6.8 ± 3.5	4.6 ± 3.4	2.9 ± 2.5	5.4 ± 2.6	43.1 ± 21.0
Cycle 2, day 1, 150 mg/m^2^ (*n* = 6)	3.3 ± 2.9	6.4 ± 5.4	4.3 ± 2.7	3.1 ± 2.8	4.5 ± 3.8	36.3 ± 30.2
Cycle 1, day 28, 200 mg/m^2^ (*n* = 1)	3	12.2	8	4.9	7.6	61.2
Cycle 2, day 1, 200 mg/m^2^ (*n* = 1)	1	14.4	5	6.7	9.8	78.4

	Total topotecan					
	*T*_max_ (h)	*C*_max_ (*μ*g/mL)	*T*_min_ (h)	*C*_min_ (*μ*g/mL)	*C*_avg_ (*μ*g/mL)	AUC_(0‐8 h)_ (h × *μ*g/mL)
Cycle 1 Day 1 – 1.0 mg/m^2^ (*n* = 3)	5.3 (±3.7)	2.4 (±1.2)	4.7 (±1.1)	50.3 (±38.0)	214.5 (±42.2)	35.9 (±17.7)
Cycle 2 Day 1 – 1.0 mg/m^2^ (*n* = 3)	3.5 (±0.4)	3.0 (±0.0)	9.2 (±3.6)	64.6 (±13.0)	106.0 (±21.0)	20.9 (±1.7)
Cycle 1 Day 1 – 1.4 mg/m^2^ (*n* = 9)	2.6 (±0.6)	2.3 (±1.0)	5.7 (±2.1)	30.6 (±9.3)	291.2 (±164.9)	75.7 (±29.8)
Cycle 2 Day 1 – 1.4 mg/m^2^ (*n* = 5)	3.0 (±0.6)	2.6 (±1.7)	11.6 (±7.2)	75.5 (±45.0)	152.7 (±87.1)	33.6 (±14.3)

	Lactone					
	*t*_1/2_ (h)	*T*_max_ (h)	*C*_max_ (ng/mL)	AUC_(0‐INF)_ (h × ng/mL)	Volume of distribution (L)	Clearance (L/h)
Cycle 1, day 1, 1.0 mg/m^2^ (*n* = 3)	4.8 ± 4.4	1.7 ± 1.2	0.6 ± 0.1	5.8 ± 5.1	1564.8 ± 211.5	341.4 ± 186.5
Cycle 2, day 1, 1.0 mg/m^2^ (*n* = 3)	3.5 ± 1.1	1.7 ± 1.2	1.6 ± 1.1	10.2 ± 8.0	951.3 ± 731.7	195.6 ± 129.0
Cycle 1, day 1, 1.4 mg/m^2^ (*n* = 9)	3.1 ± 1.4	2.3 ± 1.4	2.8 ± 1.6	14.5 ± 7.5	834.3 ± 533.4	199.4 ± 133.1
Cycle 2, day 1, 1.4 mg/m^2^ (*n* = 5)	2.0 ± 0.6	2.6 ± 1.7	6.5 ± 4.9	32.7 ± 15.1	195.2 ± 53.5	72.6 ± 23.6

For sorafenib data, 9 patients were evaluable for pharmacokinetic parameter estimate determination on cycle 1, day 28 and only 7 on cycle 2, day 1. For topotecan data, 2 patients on cycle 2, day 1 were nonevaluable for *t*_1/2_, AUC, volume of distribution, and clearance. Additionally, 4 patients did not reach cycle 2, day 1 and therefore were nonevaluable for determining all pharmacokinetic parameter estimates.

For topotecan, we evaluated 12 patients on cycle 1, day 1 and 8 patients who remained on study through cycle 2, day 1 for PK studies. The concentration‐time profiles for topotecan when dosed alone and then in combination with sorafenib were very similar (Fig. 1B and C and Table 4). We also examined PK interactions between the 2 agents to determine whether there was any increase or decrease in exposure to the substrate in the presence of the interacting drug. We assessed such interactions by AUC with its 90% confidence intervals, compared to those of their reported single agent PK values 12, 28. The 90% confidence intervals for the geometric mean ratio of the AUC for both topotecan and sorafenib throughout this study were significantly higher or at least equivalent to those of each of the two agents when given alone previously.

## Discussion

We describe the toxicity and a recommended phase 2 dose of a combination of a tyrosine kinase inhibitor and a topoisomerase I inhibitor delivered orally in children. Overall, the sorafenib and topotecan combination was tolerated with sorafenib dosed continuously at 150 mg/m^2^ by mouth twice per day and topotecan 1.4 mg/m^2^ by mouth daily. Both of these doses are 1 level below the single‐agent recommended phase II dose from prior studies 12, 28.

Although there were no unexpected side effects, we were not able to deliver either the maximum single‐agent dose for either agent, mainly due to hematologic toxicity, which precluded further dose escalation. This finding is similar to an adult ovarian tumor study 25. However, in an irinotecan and sorafenib study in metastatic colorectal carcinoma, promising clinical activity was shown with the combination even though neither medication could be delivered at the maximum single‐agent dose 29. A trial in leukemia patients concluded that sorafenib combination therapy was tolerable with efficacy when combined with nucleoside analogs 11. A goal of our trial, with oral dosing, was to keep patients at home; however, we had 3 patients admitted to the hospital for fever and neutropenia. Additionally, angiogenesis targeting tyrosine kinase inhibitors in pediatric patients with pre‐existing cardiomyopathy is challenging. Careful consideration of inclusion and exclusion criteria should be made for patients at high risk for or with known cardiomyopathy or patients on cardiac medications.

We were able to assess the majority of patients for PK. The most common reason to not have matching sets of PK parameter estimates was due to sorafenib drug interruption toward the end of cycle 1 largely due to hematologic toxicity. Our PK findings indicate large inter‐patient variability for sorafenib when dosed alone or in combination with topotecan, as shown in other studies 12, 28. Our PK results for sorafenib when used in combination were similar to a previous sorafenib single‐agent phase I study 12. For topotecan, PK profiles determined in the presence of and in the absence of sorafenib indicated no apparent drug–drug interaction. Because all outside imaging examinations were reviewed centrally, we achieved 100% concordance of determination of response per RECIST 1.1.

Although our study patients had a diversity of diagnoses, most had advanced sarcomas. Most patients entered this trial within 2 months of their most recent chemotherapy, suggesting progressive disease through the previous treatment regimen. This combination was not particularly effective in this context of chemotherapy refractory disease. In particular, in our small set of osteosarcoma patients, the activity was inferior to the recently reported sorafenib and everolimus study conducted in unresectable osteosarcoma patients 30. The therapy did offer the benefit of completely oral administration and was well tolerated.

The 2 fibromatosis patients on our trial both had been heavily pretreated and had potentially life‐threatening disease with further progression. Phase I therapy for fibromatosis has led to a promising therapy currently being evaluated in the phase II setting with PF‐03084014 31. Additionally, a single‐institution study reported promising activity of single‐agent sorafenib in fibromatosis, which provided the basis for an ongoing phase III study (NCT02066181) 32. Others have proposed that tolerability and acceptable toxicity for benign conditions be reconsidered for sorafenib 14. We report an ongoing response in a single patient and another patient with stable disease followed by radiographic progression after cycle 4.

The concept of combining tyrosine kinase inhibition with a topoisomerase inhibitor, or in more general terms targeted therapy along with cytotoxic chemotherapy, continues to be an attractive model and would likely be improved by matching to populations likely to benefit from both therapies. A more focused histology‐specific or biomarker‐specific approach could be coupled to trials to improve efficacy. A recently published model of cancer predicted more durable responses to targeted therapy when combined with a more broad inhibitor of cellular proliferation 33.

Based on the lack of a clinical signal in our study population, future development for sorafenib and topotecan may be limited unless basic and translational research yield additional insight to study the combination in a particular histology. Sorafenib has not demonstrated enough single‐agent activity in pediatric tumors or sarcomas in general and will likely need to be combined with other therapies to extend its indications 34, 35, 36. Biomarkers, or matching increasingly available mutational panels, could potentially improve patient selection for future trials with these agents. Subcellular localization of topoisomerase could also be used to predict topotecan resistance. Based on the achievable serum levels and the associated toxicities, it is unlikely that this combination at the MTD could be combined with additional hematologic toxic agents going forward.

## Conflict of Interest

None declared.

## Supporting information


**Table S1.** Time to progression (TTP) on study and TTP on prior therapy for all enrolled patients.
