# Supplementary material for: Pediatric phase I trial of oral sorafenib and topotecan in refractory or recurrent pediatric solid malignancies
Source: Cancer Med. 2015 Dec 29;5(2):294–303. doi: 10.1002/cam4.598 (PMC4735769; doi:10.1002/cam4.598)
Supplement: Supplementary file 1 — Table S1. Time to progression (TTP) on study and TTP on prior therapy for all enrolled patients. [file CAM4-5-294-s001.docx]

| **Supplemental Table 1. Time to Progression on Study and Time to Progression on Prior therapy for all Enrolled Patients** | | | | | | | |
| --- | --- | --- | --- | --- | --- | --- | --- |
| **Diagnosis** | **Days on study** | **Days on prior** | **Prior lines** | **TTP study > TTP prior?** | **Days from last therapy to enroll** | **Refractory** | **Dose level** |
| Ewing sarcoma | 57 | 108 | 3 | N | 45 | Y | 1 |
| Ewing sarcoma | 13 | 44 | 3 | N | 39 | Y | 2 |
| Ewing sarcoma | 111 | 237 | 4 | N | 202 | N | 2 |
| Osteosarcoma | 40 | 1356 | 1 | N | 1947 | N | 1 |
| Osteosarcoma | 45 | 175 | 2 | N | 50 | Y | 1 |
| Osteosarcoma | 14 | 186 | 2 | N | 479 | N | 3 |
| Osteosarcoma | 27 | 89 | 2 | N | 41 | Y | 2 |
| Fibromatosis | 135+ | 194 | 2 | Y | 158 | N | 3 |
| Fibromatosis | 104 | 21 | 4 | Y | 24 | Y | 2 |
| Neuroendocrine carcinoma | 42 | 152 | 1 | N | 29 | Y | 2 |
| Embryonal Rhabdomyosarcoma | 44 | 596 | 2 | N | 153 | N | 2 |
| Neuroblastoma | 11 | 45 | 3 | N | 63 | Y | 3 |
| **Median** | 43 | 163.5 |  |  | 56.5 |  |  |
| **Mean** | 62 | 265 |  |  | 269 |  |  |
